# Impact of the White Collar Photoreceptor WcoA on the *Fusarium fujikuroi* Transcriptome

**DOI:** 10.3389/fmicb.2020.619474

**Published:** 2021-01-18

**Authors:** Javier Pardo-Medina, Gabriel Gutiérrez, M. Carmen Limón, Javier Avalos

**Affiliations:** Department of Genetics, Faculty of Biology, University of Seville, Seville, Spain

**Keywords:** *Fusarium*, white collar protein, photoreceptor, photoregulation, secondary metabolism, carotenoids

## Abstract

The proteins of the White Collar 1 family (WC) constitute a major class of flavin photoreceptors, widely distributed in fungi, that work in cooperation with a WC 2 protein forming a regulatory complex. The WC complex was investigated in great detail in *Neurospora crassa*, a model fungus in photobiology studies, where it controls all its major photoresponses. The fungus *Fusarium fujikuroi*, a model system in the production of secondary metabolites, contains a single WC-1 gene called *wcoA*. The best-known light response in this fungus is the photoinduction of the synthesis of carotenoids, terpenoid pigments with antioxidant properties. Loss of WcoA in *F. fujikuroi* results in a drastic reduction in the mRNA levels of the carotenoid genes, and a diversity of morphological and metabolic changes, including alterations in the synthesis of several secondary metabolites, suggesting a complex regulatory role. To investigate the function of WcoA, the transcriptome of *F. fujikuroi* was analyzed in the dark and after 15-, 60- or 240-min illumination in a wild strain and in a formerly investigated *wcoA* insertional mutant. Using a threshold of four-fold change in transcript levels, 298 genes were activated and 160 were repressed in the wild strain under at least one of the light exposures. Different response patterns were observed among them, with genes exhibiting either fast, intermediate, and slow photoinduction, or intermediate or slow repression. All the fast and intermediate photoresponses, and most of the slow ones, were lost in the *wcoA* mutant. However, the *wcoA* mutation altered the expression of a much larger number of genes irrespective of illumination, reaching at least 16% of the annotated genes in this fungus. Such genes include many related to secondary metabolism, as well as others related to photobiology and other cellular functions, including the production of hydrophobins. As judged by the massive transcriptomic changes exhibited by the *wcoA* mutant in the dark, the results point to WcoA as a master regulatory protein in *F. fujikuroi*, in addition to a central function as the photoreceptor responsible for most of the transcriptional responses to light in this fungus.

## Introduction

The genus *Fusarium* comprises a large group of ubiquitous fungal species of great economic importance, frequently associated with mycotoxin production and plant pathogenesis (Geiser et al., 2013). Many *Fusarium* strains stand out for their capacity to produce a large array of secondary metabolites (SMs). A representative example is *Fusarium fujikuroi* (Janevska and Tudzynski, 2018), a rice pathogen known for its ability to produce gibberellins (Studt and Tudzynski, 2014), plant hormones of the terpenoid family that are used in agriculture due to their stimulatory effects on plant growth and development. This fungus is capable of producing many other metabolites (Niehaus et al., 2017), which include other terpenoids, as carotenoids, and various polyketides, such as bikaverin (Studt and Tudzynski, 2014) and fusarins (Niehaus et al., 2014). Carotenoids are lipophilic pigments produced by all photosynthetic organisms and by many heterotrophic bacteria and fungi (Concepcion et al., 2018). *Fusarium* sp. produces an orange carotenoid, neurosporaxanthin (NX), which provides a characteristic orange pigmentation to its colonies. In recent years, *F. fujikuroi* has become a model in the synthesis of these pigments in fungi, and all the genes specifically involved in its carotenoid metabolism have been characterized (Avalos et al., 2017). Two key enzymes in the pathway, *carRA* and *carB*, are involved in the synthesis of the precursor phytoene and the subsequent desaturation and cyclization reactions necessary for the production of colored carotenoids. Both genes are linked to the carotenoid oxygenase gene *carX* and the rhodopsin gene *carO* in a coregulated cluster, while other genes of the pathway are spread in other locations in the genome.

Light is an important regulatory cue in fungi (Fischer et al., 2016). Secondary metabolism is normally not greatly affected by light in *Fusarium*; however, light is an important regulatory signal in the control of carotenogenesis (Avalos and Estrada, 2010). Early studies in *F. aquaeductuum* showed that illumination of mycelia grown in the dark results in the accumulation of carotenoids to reach its maximum after 12 h of exposure to light, and the analysis of the action spectrum for NX photoinduction suggested the participation of a flavin photoreceptor (Rau, 1967). Similar patterns of NX photoinduction and action spectrum were previously described in *Neurospora crassa*. In both fungi, photoinduction occurs at the transcription level, with rapid mRNA formation after illumination. In *N. crassa*, this and other photoresponses are mediated by the White Collar complex (WCC), a heterodimer formed by the WC-1 photoreceptor and its partner WC-2 (Fischer et al., 2016). Therefore, the WC-1 and WC-2 mutants of *N. crassa* cannot accumulate carotenoids in response to light in mycelia (Degli-Innocenti and Russo, 1984). The central regulatory role is carried out by WC-1, which binds a flavin through its LOV (Light, Oxygen, Voltage) domain. Light absorption by this flavin results in a conformational change in WC-1 that triggers WCC activation. The molecular mechanism of the WCC has been investigated in great detail. Light-activated WCC allows the dimerization of two WCCs through the LOV domains of two WC-1 subunits, making it a transcription factor capable of binding light-responsive elements in the promoters of target genes (Schafmeier and Diernfellner, 2011; Corrochano, 2019). Activation by the WCC is transient, since it is subsequently inactivated by the small flavoprotein VVD, whose gene was in turn induced by the activated WCC, giving rise to a mechanism known as photoadaptation (Chen et al., 2010a; Malzahn et al., 2010). In addition, the WCC plays an important role as the main transcription factor that controls the circadian clock in *N. crassa*, directly and indirectly driving the rhythmic expression of hundreds of genes (Sancar et al., 2015), a process in which participates the protein FRQ (Cha et al., 2015). Although its regulatory activity includes synchronization of the cycle to light-dark regimes, WCC-modulated circadian rhythmicity is maintained in continuous darkness, indicating a light-independent activity of the WCC complex.

Fungal genomes frequently contain orthologs for *wc-1* and *wc-2* genes, and this is the case of *Fusarium* (Corrochano, 2019). Due to its taxonomic proximity to *Neurospora* and the occurrence of photoinduced NX accumulation, an albino phenotype was predictable in equivalent *wc-1*-like *Fusarium* mutants. However, null mutants of the *wc-1* orthologous genes of *F. fujikuroi* (*wcoA*) and *F. oxysporum* (*wc1*) exhibited accumulation of carotenoids under illumination (Estrada and Avalos, 2008; Ruiz-Roldán et al., 2008), suggesting the participation of at least one additional photoreceptor. However, WcoA is the major transcriptional activator of the genes of the carotenoid pathway of *F. fujikuroi*, as indicated by the drastic reduction of mRNA levels for the *carRA* and *carB* genes in the absence of this protein, either in darkness or after illumination (Estrada and Avalos, 2008; Castrillo and Avalos, 2015). Furthermore, the syntheses of other metabolites, such as bikaverin, gibberellins or fusarins, were also altered in the *wcoA* mutants, which points to a broader regulatory role in SM production (Estrada and Avalos, 2008). Interestingly, the mutation causes phenotypic effects not only under light but also in darkness, and affects other biological processes, such as morphology, surface hydrophobicity, and conidiation, indicating that WcoA is capable of controlling the transcription of many genes in the absence of its alleged regulatory signal. To gain information on the functions in *Fusarium* of this widespread fungal photoreceptor, we describe here the effect of the *wcoA* mutation on the *F. fujikuroi* transcriptome, either in the dark or after several times of illumination.

## Materials and Methods

### Strains and Culture Conditions

*Fusarium fujikuroi* FKMC1995 (Kansas State University Collection, Manhattan, KS, United States) was used as the wild strain. The *F. fujikuroi* Δ*wcoA* mutant SF226 was generated by interruption of the *wcoA* coding region with a hygR resistance cassette (Estrada and Avalos, 2008). Strains were cultured in DG minimal medium except for conidia production, in which case they were grown in EG medium for 7 days (Marente et al., 2020). For expression studies, the strains were grown in 100 ml of DG medium in 500 ml Erlenmeyer flasks, inoculated with 10^6^ conidia. The flasks were kept in total darkness for 3 days at 30°C in an orbital shaker (150 rpm). Then, the cultures were transferred to four 25-ml Petri dishes under safe red light and kept in static conditions in the dark for 240 min. Subsequently, the Petri dishes were illuminated for the indicated times, from 15 min to 240 min or incubated in the dark. Illumination was provided by a set of four fluorescent tubes (Philips TL-D 18 W/840) at a distance of 60 cm, yielding a light intensity of 7 Wm^–2^. Mycelial samples were collected by filtration and frozen immediately at −80°C for future use.

### Expression Studies

Total RNA was extracted from 150 to 200 mg of ground mycelia samples with Trizol (Invitrogen, Paisley, United Kingdom), using the protocol described by the manufacturer. RNA concentrations were quantified with a Nanodrop ND-1000 spectrophotometer (Nanodrop Technologies, Wilmington, DE, United States). For qPCR measurements, 2.5 μg RNA samples were treated with DNAse I and retrotranscribed to cDNA with Transcriptor first-strand cDNA synthesis kit (Roche, Mannheim, Germany). Final cDNA concentrations were adjusted to 25 ng/μl. RT-qPCR analyses were performed in a LightCycler 480 real-time instrument (Roche) using LightCycler 480 SYBR Green I Master (Roche) following manufacturer reaction protocol. Genes and primer sets (forward vs. reverse in 5′- > 3′ orientation and amplicon length) were *carRA* (CAGAAGCTGTTCCCGAA- GACA vs. TGCGATGCCCATTTCTTGA, 65 bp) and *carB* (TCGGTGTCGAGTACCGTCTCT vs. TGCCTTGCCGGTT- GCTT, 68 bp). Transcript levels for each gene were normalized against the tubulin beta chain gene FFC1_09881 (CCGGTGCTGGAAACAACTG vs. CGAG GACCTGGTCG- ACAAGT, 69 bp). The data were relativized to the value of the wild strain grown in the dark, which was taken as 1. The statistical significance of differences between mRNA values and reference value was verified with the one sample *t* test, using Graphpad Prism version 8.0.2 for Windows (GraphPad Software, San Diego, CA, United States).

### RNA-Seq Methodology

A total of 20 μg of each RNA sample were treated with DNAse using columns from the NucleoSpin RNA kit (Macherey-Nagel, Düren, Germany) following manufacturer’s instructions. Quality parameters of absorbance (Ratio A260/A280 > 1.8 and A260/A230 > 1.5, and RIN > 8.5) were tested before the processing of the samples with the Agilent “Chip RNA Plant Nano” protocol by the company Life Sequencing (Valencia, Spain). Samples were sequenced on Illumina’s NextSeq platform in 75 bp single read mode. “Bcl2fastq2” version 2.19.1 provided by Illumina was used for the conversion of “bcl” files into “fastq” sequence files, a program that also removes the sequencing adapters. Quality values, number of readings and total sequences, are shown in Supplementary Table 1.

### Bioinformatic Analyses

The experiment includes three samples per strain and condition. Raw reads for all samples were trimmed, filtered and quality controlled with AfterQC (Chen et al., 2017). Sequences were mapped with TopHat 2.1.1 (Trapnell et al., 2009). The Integrative Genomics Viewer IGV application (IGV) version 2.8 was used for mapping visualization (Robinson et al., 2011). A meta-assembly of the transcriptome with the Cufflinks-Cuffmerge protocol (Roberts et al., 2011) was generated to improve the level of annotation of the analyzed strain. Mapped sequences were analyzed using SeqMonk (version 1.45.4^1^). Quantification was performed using the RNAseq quantitation pipeline with the improved mRNA annotation generated by Cuffmerge, merging transcripts and counting reads over exons. Deseq2 tool (Love et al., 2014), implemented in SeqMonk, which needs raw counts for quantitation, was used to compare among conditions. The differentially expressed genes were selected based on criteria combining a log2 fold change of 2 and a *p*-value of 0.05. Log2 RPM (reads per feature per million reads of library) were used for data visualization and intensity tests, which had minimum *p*-value below 0.05 multiple testing correction applied with a sample size of 100 when constructing the control distributions. Venny 2.1 was used to draw Venn Diagrams. Gene expression levels were measured as TPM (transcripts per million) to represent the heatmaps. Heatmap figures were performed using the mean between the three samples in TPM for each strain and condition, then data were log transformed and centered using the mean between them, and hierarchically clustered with Gene Cluster 3.0 (de Hoon et al., 2004). The visualization was performed with Java TreeView3 (Saldanha, 2004).

To perform GO enrichment analysis, the *F. fujikuroi* FKMC1995 proteome was annotated using the corresponding IMI58289 orthologs annotation data from Fungi DB. To assign orthologs a formerly proposed equivalence was used (Niehaus et al., 2017). Go terms corresponding to each subset of genes were extracted and tested with a *p*-value cut off of 0.05. Computed and curated terms were used in the analysis. FunCat GO enrichment results were obtained from the FungiFun database^2^ using a significance level of 0.05 and testing for enrichment with FDR correction.

Orthologs of *F. fujikuroi* for the clock-controlled genes whose expression was affected in *csp-1* mutant in *N. crassa* (Sancar et al., 2015) were obtained from the FungiDB database (Basenko et al., 2018). Orthologs were found for the wild strain IMI58289, and FKMC1995 counterparts were obtained as described in section “Functional Categories of Genes Influenced by Light in *F. fujikuroi*.”

## Results

### Experimental Design

**FIGURE 1:**
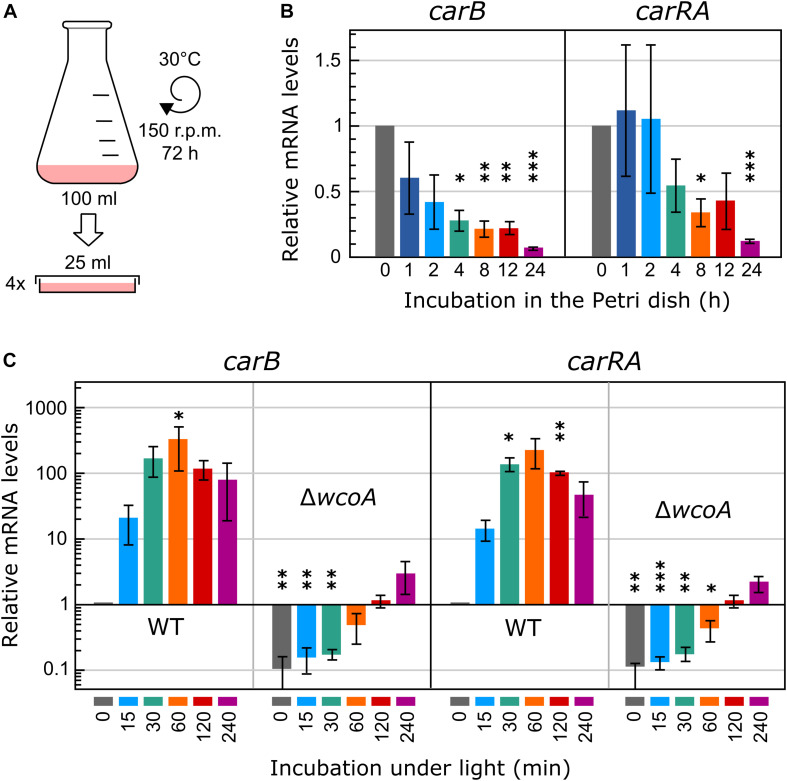
Experimental design and mRNA levels of the *carRA* and *carB* genes. **(A)** Schematic representation of the experimental conditions used for illumination. The cultures were transferred to Petri dishes to improve homogeneous illumination. **(B)** Effect of the incubation of the mycelia of the wild strain in the Petri dish before illumination on mRNA levels of the *carRA* and *carB* genes. **(C)** Effect of illumination on the mRNA levels of the *carRA* and *carB* genes after 4-h incubation in the Petri dish in the dark. In **(B)** and **(C)** relative mRNA levels are referred to mRNA content of the wild strain in darkness, and differences with this value found to be significant according to the *t* tests are indicated (*p*-values, ^∗^*p* < 0.033; ^∗∗^*p* < 0.002; ^∗∗∗^*p* < 0.001).

In a recent study, we investigated the effect of light on the *Fusarium* transcriptome by comparing total RNA samples from mycelia illuminated for 60 min with mycelia non-illuminated (Ruger-Herreros et al., 2019). This time of illumination had been chosen because in previous RT-PCR studies it resulted in the highest mRNA levels for the carotenoid genes (Castrillo and Avalos, 2015). In our former transcriptomic analysis, the submerged mycelia from each flask were immediately transferred to four Petri dishes prior to illumination to facilitate homogeneous light exposure to the cultures (Figure 1A). Here, to analyze more precisely the role of WcoA, different illumination times have been tested. Since the transfer implies a change in the aeration conditions, the effect of this alteration was first checked for the mRNA levels of the carotenoid genes, used here as a reference for light-induced expression. As a result, a reduction of the mRNA levels of *car* genes was observed during the first 4 h (Figure 1B). Therefore, for a better comparison of different light exposures, illumination was carried out in this study after 4 h of adaptation of the culture to the static conditions in the Petri dish in the dark. The effect of different illumination times, 15, 30, 60, 120, and 240 min, was analyzed under these conditions for the *carRA* and *carB* genes (Figure 1C). As expected, the mRNA content increased rapidly upon illumination and reached maximal values after 60 min, to slowly decrease afterward. Parallel cultures with the *wcoA* mutant revealed a drastic decrease in the mRNA levels of the same genes (Figure 1C), but they exhibited a noticeable increase upon illumination for 60 min or longer. Taking these data into account, to investigate the role of the WcoA photoreceptor at the global transcriptomic level, RNA-seq analyses were performed with mycelia of the wild strain and the *wcoA* mutant SF226 incubated either in the dark or exposed to 15, 60, and 240 min of illumination.

### Effect of Illumination Time in the Wild Strain Transcriptome

**FIGURE 2:**
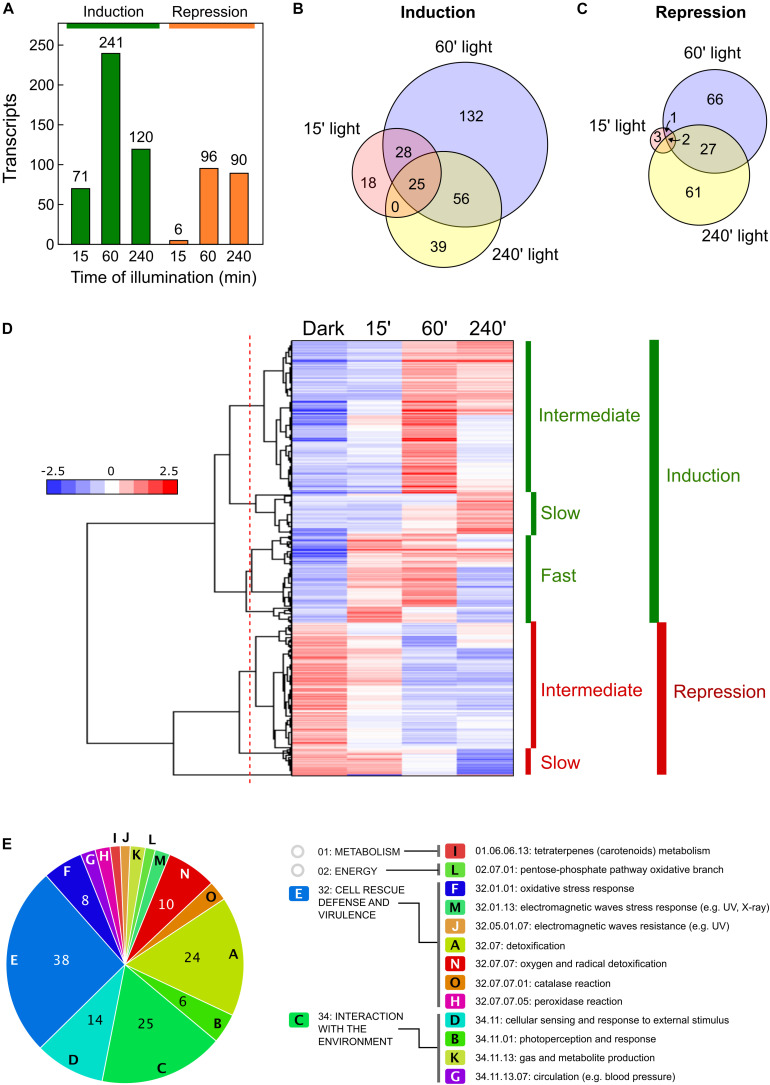
Effect of illumination on the wild *F. fujikuroi* transcriptome. **(A)** Numbers of genes upregulated (green) or downregulated (orange) after 15-, 60-, or 240-min illumination. **(B,C)** Venn diagrams of the overlap between the genes induced **(B)** or repressed **(C)** after the illumination times indicated. **(D)** Hierarchical heatmap of the genes induced or repressed under the illumination times indicated. **(E)** Funcat categories of the genes induced after 60-min illumination.

Genes, whose transcript levels changed at least four-fold (log2 = 2) after illumination were considered as differentially expressed. Using this criterium, the numbers of genes differentially expressed in the wild strain after the different illumination times are indicated in Table 1. Among those influenced by light, our analyses revealed new transcripts that did not correspond to genes formerly identified in the genome annotation (indicated in parenthesis in Table 1). Total numbers of differentially expressed transcripts after different illumination times are represented in Figure 2A. Details on the lists of genes are provided in Supplementary Table 2. The major effect of light was observed after 60 min illumination, resulting in changes in expression of 337 transcripts. There was a predominance of induction over repression (*ca.* 71% vs. 29%), and a higher impact on change magnitudes in the induced genes: 17 transcripts exhibited more than 64-fold increase (log2 = 6) after 60-min illumination compared to the control in the dark, while the highest repression was of log2 = −5.8 (Supplementary Table 2). Impact of light was less severe after 15-min or 240-min illumination (compare scatter plots in Supplementary Figures 1A,C,E), with only 77 and 210 transcripts with changes exceeding the four-fold threshold (Figure 2A). Induction was predominant in the first 15 min of illumination, *ca.* 92%, but this proportion decreased to *ca.* 57% after 240 min, probably reflecting secondary transcriptional effects.

**TABLE 1:** Number of genes whose expression changes more than four-fold (log2 = 2) above (activated) or below (repressed) the controls, under the indicated conditions.

	Activated	%	Repressed	%
Effect of 15 min light in the wild strain	67 (+4)	0.44	5 (+1)	0.03
Effect of 60 min light in the wild strain	226 (+15)	1.50	84 (+12)	0.56
Effect of 240 min light in the wild strain	116 (+4)	0.77	80 (+10)	0.53
Effect of 15 min light in the *wcoA* mutant	1	<0.01	0	–
Effect of 60 min light in the *wcoA* mutant	3 (+1)	0.02	0	–
Effect of 240 min light in the *wcoA* mutant	21 (+5)	0.14	22	0.15
Effect of *wcoA* mutation in the dark ^a^	588 (+86)	3.90	1877 (+253)	12.43
Effect of *wcoA* mutation after 15 min light ^a^	750 (+116)	4.97	1567 (+220)	10.38
Effect of *wcoA* mutation after 60 min light ^a^	708 (+102)	4.69	1870 (+205)	12.39
Effect of *wcoA* mutation after 240 min light ^a^	560 (+101)	3.71	1530 (+164)	10.14

*The percentage refers to the total number of genes annotated in the reference genome (15095). New transcripts not formerly annotated are indicated in parenthesis. ^*a*^ Comparisons between wild strain and *wcoA* mutant.*

Overall, 298 transcripts were induced by light under any of the illumination conditions tested. However, they exhibited considerable variation in induction patterns. This was clearly indicated on a Venn diagram by the partial overlap between those induced after 60 min under light with those induced after 15 min or 240 min, with only 25 transcripts induced at the three illumination times (Figure 2B). Like so, of the 241 transcripts induced after 60 min of light, 132 were not induced at the other exposure times, and just a few were only induced after 15 min (18), or after 240 min (39). Variability of responses is best visualized on a hierarchical clustering heat map (Figure 2D). This graphical representation shows that light stimulation is frequently transient, with highest induction after 60 min under light (called intermediate induction in the graph). There were 161 genes in this category (detailed clustering data in Supplementary Table 3). There were variations among them in the level of adaptation to longer illumination, with higher or lower decreases after 240 min compared to levels at 60 min. Two other groups could be distinguished in the clustering: a set of 94 transcripts exhibiting rapid induction after 15 min (fast induction), and a set of 43 transcripts that reached their higher levels after 240 min (slow induction).

Less variability was observed in the transcripts repressed by light, with a higher incidence of genes repressed after longer light exposures (Figure 2C). Clustering analysis (Figure 2D) revealed two major sets of transcripts, consisting of 132 that exhibited significant repression after 60 min of light (intermediate repression) and 27 genes with a sharp decrease in mRNA levels only after 240 min of illumination (slow repression). Transcripts with a sharp reduction in the first 15 min of illumination were essentially absent.

The results after 60-min illumination were comparable to those obtained with the wild strain IMI58289 (Ruger-Herreros et al., 2019), in which log2 = 1 was applied as the induction/repression threshold. In that case, the Cufflinks method was used instead of the stricter SeqMonk protocol. If the Cufflinks method was applied to our not-illuminated and 60-min-illuminated samples in the wild strain FKMC1995, with the log2 = 1 threshold, the results revealed 920 upregulated and 590 downregulated genes, similar to the 724 and 535 genes described in the equivalent samples in the wild strain IMI58289 (Ruger-Herreros et al., 2019).

### Functional Categories of Genes Influenced by Light in *F. fujikuroi*

As formerly done for the effect of light on another *F. fujikuroi* strain (Ruger-Herreros et al., 2019), GO enrichment analyses were performed to study the effect of light on *F. fujikuroi* FKMC1995 mycelium. To identify the GO terms of its proteome we used the only annotation available for this strain (Niehaus et al., 2017), and the correspondence with the orthologs in *F. fujikuroi* IMI 58289 (genomic locations and equivalence data shown in Supplementary Table 4). FunCat data on significant functional categories revealed reliable results only for the set of genes induced by light for 60 min (Figure 2E). The list included 154 genes, belonging to 15 major categories and subcategories involved in very diverse metabolic functions (Supplementary Table 5), including the one of metabolism related with carotenoid biosynthesis. Most of them belong to two major groups “Cell rescue defense and virulence” and “Interaction with the environment.” The first one included some genes encoding proteins homologous to enzymes with presumable detoxification functions, such as a thioredoxin (FFC1_12309, log2 fold change 2.2), a thioredoxin peroxidase (FFC1_05108, log2 = 2.6), a glutathione S-transferase II (FFC1_12341, log2 = 2.7), an organic hydroperoxide resistance protein (FFC1_02122, log2 = 5.0) and two catalases (FFC1_14936, log2 = 2.4, and FFC1_13476, log2 = 3.2). The second one included a putative heat shock protein of the HSP30 family (FFC1_12352, log2 = 8.3), and some putative photoreceptors that remain to be investigated in this fungus, one similar to the phototropin “non-phototropic hypocotyl protein 1” (FFC1_12444, log2 = 3.2), and a deoxyribodipyrimidine photo-lyase gene (FFC1_07528, log2 = 2.2), actually encoding a putative plant cryptochrome. Two other genes encoding proteins belonging to the deoxyribodipyrimidine photo-lyase group in *Fusarium*, the DASH cryptochrome CryD and the photolyase PHR, were detected as significant with this methodology only in the group of the early expressed genes, although they also exhibited a very strong photoinduction after 60 min of illumination (*cryD*: FFC1_04237, log2 = 8.9; *phr*: FFC1_00478, log2 = 5.2).

The FunCat results were less consistent for genes induced after 15 min or 240 min, or for genes repressed by light. A parallel analysis performed for the entire set of light-induced genes, grouping those induced after 15, 60 or 240 min of light, provided results similar to those obtained with 60 min, but in this case the total number of categories raised to 19 (Supplementary Figure 2).

### Effect of Light in the *wcoA* Mutant Transcriptome

**FIGURE 3:**
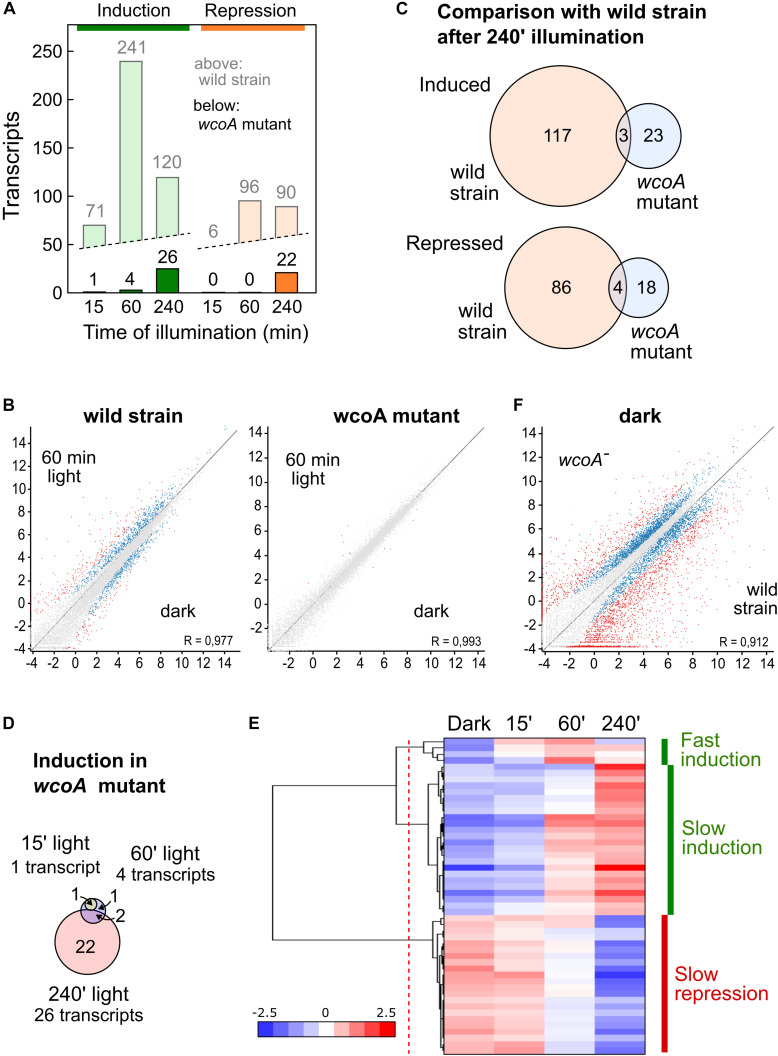
Effect of illumination on the transcriptome of the *wcoA* mutant. **(A)** Numbers of genes upregulated (green) or downregulated (orange) after 15, 60, or 240 min illumination. For comparison, the data for the wild strain are shown in pale color. **(B)** Scatter plot representations (log2 rpm values) of the effect of 60 min illumination in the wild strain and in the *wcoA* mutant. Genes differentially expressed according to the Deseq analysis of the SeqMonk program are indicated in blue. Genes exceeding the log2 values of + – 2 are indicated in red. **(C)** Venn diagrams of the genes induced (above) or repressed (below) after 240 min illumination in the wild strain and in the *wcoA* mutant. The intersection shows the number of genes induced or repressed in both strains. **(D)** Venn diagrams of the genes induced in the *wcoA* mutant after the indicated illumination time. **(E)** Hierarchical heatmap of the genes induced or repressed in the *wcoA* mutant under the indicated illumination times. **(F)** Scatter plot representation of the effect of the *wcoA* mutation in the dark. Details as described in **(B)**.

The RNA-seq data showed a drastic reduction in the number of transcripts affected by light in the *wcoA* mutant (Figure 3A). This is clearly visible in comparisons of scatter plots with those of the wild strain (data for 60-min illumination in Figure 3B, results for the three illumination times shown in Supplementary Figures 1A–F; the genes affected by light in the *wcoA* mutant are listed in Supplementary Table 6). None of the transcripts induced or repressed after 15- or 60-min of illumination in the wild strain were significantly affected by light in the *wcoA* mutant, and the few genes induced by light in the mutant after these light exposures were not affected by light in the wild strain after the same illumination. Moreover, 26 and 22 genes were, respectively, induced and repressed in the *wcoA* mutant after 240 min of light exposure (Figure 3A), with very few coincidences with the equivalent gene sets in the wild strain (Figure 3C). Taken together, the data point to WcoA as the major photoreceptor responsible for photoregulation of gene expression in *F. fujikuroi*.

Only four genes were induced by light in the *wcoA* mutant after 15 min or 1 h, with a highest induction ratio of log2 = 3.4 (FFC1_15159, coding for an uncharacterized protein), and only two of them were also present in the set of 22 genes induced after 240 min (Figure 3D). The pattern of transcript levels for the 26 genes induced by light was more homogeneous in the *wcoA* mutant (Figure 3E), with a clear separation between 4 genes exhibiting a relatively fast response (Intermediate induction) and 24 exhibiting a slower response (Slow induction, detailed clustering data in Supplementary Table 7). However, the 22 genes that were repressed by light in the mutant responded slowly, with significant reductions only after 240 min of illumination (Slow repression).

The 24 transcripts induced by 240 min of light in the *wcoA* mutant included those for *carRA* and *carB*, already found to show photoinduction in our RT-PCR assays of this strain (Figure 1C). This set also included genes for at least five proteins presumably associated to stress control: FFC1_04281 (related to multidrug resistance protein), FFC1_01433 (probable heat shock protein 30), FFC1_09886 (related to AHA1-stress-regulated cochaperone), FFC1_09644 (related to ADH4-alcohol dehydrogenase IV), and FFC1_12341 (related to glutathione S-transferase II). Of these five genes, only FFC1_12341 exhibited a significant induction by light in the wild strain, which was delayed in the mutant. On the other hand, the 22 transcripts repressed after 240 min light in the *wcoA* mutant included 8 genes associated to transport through the membrane: FFC1_08950 (probable Na^+^-transporting ATPase ENA-1), FFC1_05826 (related to zinc transporter), FFC1_09497 (probable SIT1-Transporter of the bacterial siderophore ferrioxamine B), FFC1_04029 (related to peptide transport protein), FFC1_14622 (NAAP-1 amino acid permease NAAP1), FFC1_09245 (related to permeases-unknown function), FFC1_09585 (probable general amino acid permease), FFC1_15542 (probable uracil permease), as well as three other integral membrane proteins of unknown function.

### Effect of the *wcoA* Mutation in the *F. fujikuroi* Transcriptome

**FIGURE 4:**
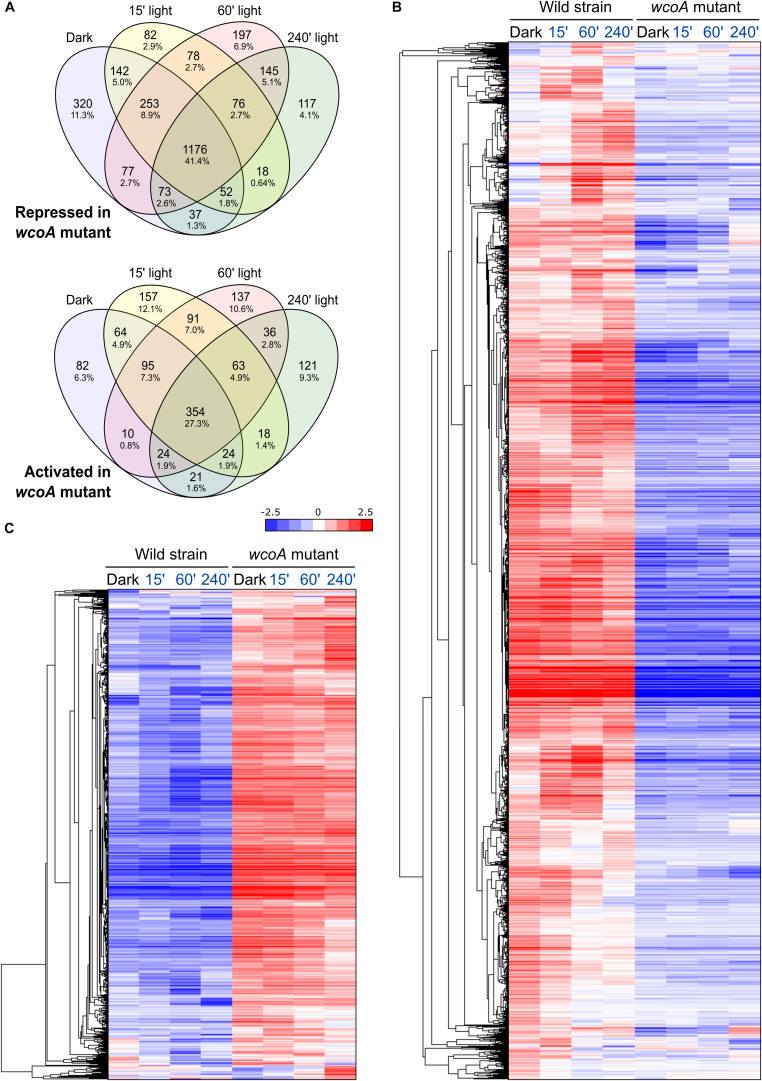
Effect of the *wcoA* mutation on the *F. fujikuroi* transcriptome under different light regimes. **(A)** Venn diagrams of the genes repressed (above) or activated (below) in the *wcoA* mutant in the dark or after the illumination times indicated. **(B,C)** Hierarchical heatmaps of the genes repressed **(B)** or induced **(C)** in the *wcoA* mutant under the illumination times indicated above.

Comparison of the transcriptome of the *wcoA* mutant with that of the wild strain, without considering illumination, revealed massive changes (Table 1). In the dark, a total of 674 and 2130 transcripts were found to be activated or repressed in the *wcoA* mutant, respectively, relative to the wild strain. Similar numbers of activated or repressed transcripts were also found after 15-, 60-, and 240-min illumination (866, 810, and 661 for activation, and 1787, 2075, and 1694 for repression, respectively). The lists of genes are described in Supplementary Table 8. Taken globally, 2843 transcripts were repressed and 1297 were activated in the *wcoA* mutant in the dark or after any of the light exposures investigated (Figure 4A). This means that 4140 transcripts, *ca*. 1/4 of the total number of annotated genes, changed at least four-fold their levels due to the *wcoA* mutation in at least one of the conditions tested. Even considering that these transcripts may include a high proportion of cascading effects, the results suggest that WcoA is an important transcription regulator in *F. fujikuroi*. Moreover, the abundance of downregulated genes in the *wcoA* mutant indicates a predominant role for WcoA as a positive regulator.

The massive effects of the *wcoA* mutation in the *F. fujikuroi* transcriptome are visually captured in scatter charts (the effect in the dark shown in Figure 3F, the effect under all conditions displayed in Supplementary Figures 1G–J) or in heat map representations (Figures 4B,C). The expression patterns of the 674 and 2130 transcripts activated or repressed in the *wcoA* mutant in the dark show that the effects are generally independent of light. Although there are variations in the transcript levels according to the illumination time, either up- or downregulation is usually maintained by most of the genes irrespective of light. Therefore, although WcoA is the key regulator involved in the control of gene expression by light, its major regulatory role is light independent.

Because the *wcoA* mutation exerts a major effect on the *F. fujikuroi* transcriptome, an alternative approach to perform differential expression analysis was used with the investigated samples. The alteration of a biological system, in this case generated either by light or by the absence of a functional WcoA protein, is expected to affect large numbers of genes as the whole system becomes destabilized. Therefore, we applied the intensity difference filter of the SeqMonk tool that checks the distribution of differences to find those whose change is not explained by the general level of disruption in the system. This method is expected to detect transcripts that changed more strongly as they directly respond to the stimulus. Since it is a stricter method, it usually reduces the list of hits. In our case, the intensity difference filter of SeqMonk detected 559 transcripts (Supplementary Table 9), which predictably explain the major transcriptomic differences in the *wcoA* mutant compared to the wild strain.

### Functional Categories of Genes Influenced by WcoA in *F. fujikuroi*

**FIGURE 5:**
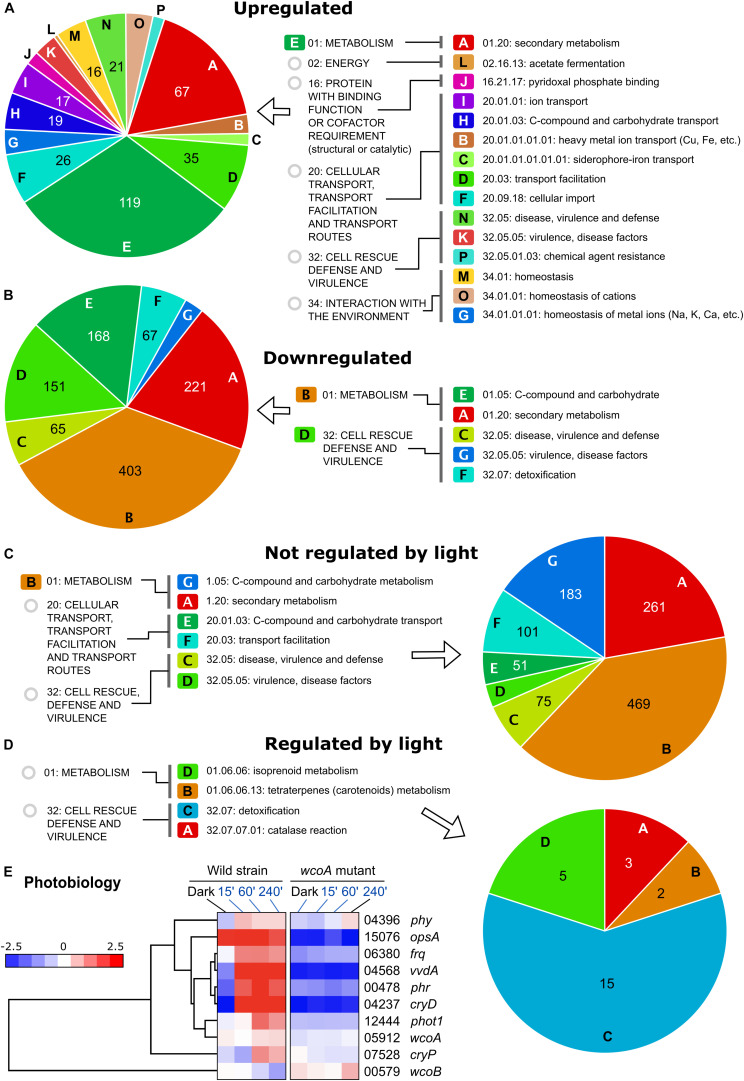
Funcat categories of the genes affected by the *wcoA* mutation. **(A)** Categories of genes induced in the *wcoA* mutant (therefore, downregulated by WcoA). **(B)** Categories of genes repressed in the *wcoA* mutant (therefore, upregulated by WcoA). **(C)** Categories of genes not regulated by light in the wild strain whose transcripts changed in the *wcoA* mutant. **(D)** Categories of genes regulated by light in the wild strain whose transcripts changed in the *wcoA* mutant. **(E)** Hierarchical heatmaps for the effect of illumination and/or the *wcoA* mutation on the mRNA levels of genes related to *F. fujikuroi* photobiology. FFC1_ numbers of the genes, and names given in the literature, are indicated on the right.

If most of the effects of the *wcoA* mutation are independent of light, data with different light exposures provide experimental repeats of such effects. For more solid statistical reliability, FunCat analyses of the WcoA-controlled genes were limited to those that changed in the mutant in all four conditions tested (dark, and 15-, 60-, and 240-min illumination). These were found to be 354 induced and 1176 repressed in the *wcoA* mutant (Figure 4A, list of genes detailed in Supplementary Table 10). Representations of the gene distributions in significant FunCat categories are displayed in Figure 5. Data were analyzed separately for those upregulated or downregulated in the *wcoA* mutant, and for those regulated or not by light in the wild strain.

The greatest diversity of functional categories was found in the set of genes with increased mRNA levels in the *wcoA* mutant, while fewer functional categories were observed in the set of downregulated genes (Figures 5A,B and Supplementary Table 5). In both cases, the most relevant FunCat classes were related to metabolism, especially those involving secondary metabolism (described in next section). Among the upregulated genes, stand out those related to transport systems (categories I, H, B, C, D, F in Figure 5A) and ion homeostasis (M, O, G). Other highly represented groups include genes of the cell rescue defense and virulence categories, suggesting a possible participation of WcoA in pathogenesis. When the response to light in the wild strain is considered (Figures 5C,D), secondary metabolism is also a predominant category in the genes not controlled by light, together with other functional categories already described in Figures 5A,B. The genes regulated by light in the wild strain, and affected by the *wcoA* mutation, include those of carotenoid metabolism and others involved in detoxification, especially the genes with catalase functions, already mentioned in the section “Functional Categories of Genes Influenced by Light in *F. fujikuroi*.”

FunCat categories were also applied to the list of 559 WcoA-influenced genes resulting after application of the SeqMonk intensity difference filter (see section “Effect of the *wcoA* mutation in the *F. fujikuroi* Transcriptome”). This reinforces the categories related to secondary metabolism and transport systems as those more relevant in the genes not regulated by light, while stress resistance and ion homeostasis were prevalent among the light-controlled genes (Supplementary Figure 3).

Photoreceptors are a heterogeneous group of proteins not identified as a FunCat category. Former data showed that the genes for the DASH cryptochrome CryD (Castrillo et al., 2013), the small flavoprotein VvdA (Castrillo and Avalos, 2014), and the photolyase Phr (Ruger-Herreros et al., 2019), are strongly photoinduced, and at least in the first two cases, their inductions are mediated by WcoA. Our RNA-seq data confirmed the dependence on WcoA of these photoinductions (Figure 5E). The *carO* rhodopsin gene, described in the next section, also belongs to this category due to its close connection with the genes of the carotenoid cluster. The second rhodopsin gene, *opsA*, is not photoinduced, but unexpectedly its expression is highly dependent on WcoA. The genes for two other predicted photoreceptors, the plant-like cryptochrome CryP and the phytochrome Phy1, exhibit a slight photoinduction, with a clear dependence on WcoA only in the case of *cryP*. Finally, the photoinduction of the phototropin gene mentioned in the section “Results,” which we call Phot1, is lost in the *wcoA* mutant.

The *wcoA* mutant phenotype includes a reduced mycelial hydrophobicity (Estrada and Avalos, 2008). A recent analysis in *F. graminearum* revealed five genes with hydrophobin domains, that were named *Fghyd1-5* (Quarantin et al., 2019). The *F. fujikuroi* genome contains orthologs for these genes, abbreviated here as *hyd1-5*. The analysis of the RNA-seq data for these genes showed that *hyd3* stood out among the others because of its very high mRNA amounts at the growth conditions tested (Supplementary Figure 4), while there were very low mRNA levels for *hyd2*, *hyd4*, and *hyd5* and no mRNA could be detected for *hyd1*. Interestingly, the amount of *hyd3* mRNA was strongly reduced in the *wcoA* mutant, while that of *hyd4* was increased, although it still remained at low levels compared to those of *hyd2*.

### Role of WcoA in Secondary Metabolism

**FIGURE 6:**
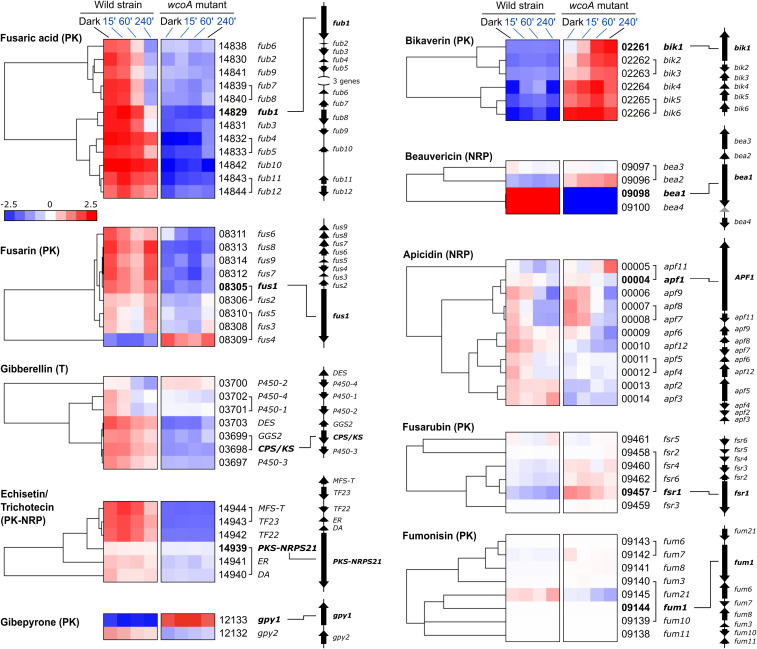
Effect of illumination and/or the *wcoA* mutation on the mRNA levels of the genes of the indicated SMs. Data are shown as hierarchical heatmaps. FFC1_ numbers of the genes and names given in the literature, are indicated on the right, together with their genomic organizations. Genes divergently transcribed are joined with a bracket. The most relevant gene in the biosynthetic pathway is indicated in bold letters.

Genes involved in the production of SMs are usually clustered in the *F. fujikuroi* genome. A detailed view in heat map representations of the effect of WcoA on SM gene clusters is displayed in Figure 6, together with the genomic organizations of the clusters. The absence of a functional WcoA protein affected the analyzed clusters in different ways. Clusters for fusaric acid, fusarin, gibberellin, and echistatin/trichothecene production were mostly downregulated in the *wcoA* mutant. This was not observed for all genes, e.g., the PKS-NRPS-21 gene, encoding the key enzyme for echistatin/trichothecene production, was barely affected by the *wcoA* mutation, and the genes of the fusarin and gibberellin clusters, FUS4 and P450-2, exhibited an opposite regulation, with larger transcript levels in the *wcoA* mutant. On the other hand, the bikaverin cluster showed the opposite regulation, with larger mRNA levels for all *bik* genes in the absence of a functional WcoA protein. Interestingly, the beauvericin and gybepirone clusters contained few genes, but with drastic regulatory differences. However, its genes for the key biosynthetic enzymes GPY1 and BEA1 showed strong WcoA regulations. In contrast, although there were minor effects in some of them, the *wcoA* mutation did not significantly affect most genes in the apicidin, fusarubin, and fumonisin clusters.

*F. fujikuroi* is predictably capable to produce a wide array of SMs, as indicates the large number of genes encoding enzymes of the NRPS, PKS, terpene cyclase families, as well as others (Supplementary Table 11). Only 7 out of 18 functional PKS genes identified in the *F. fujikuroi* genome are mentioned in Figure 6. However, three other PKS genes of unknown function (FFC1_04787, 12077, and 00016) were significantly affected by the *wcoA* mutation. This was also the case for other presumptive SM genes not mentioned in Figure 6, such as four genes for predicted NRPS enzymes (FFC1_07944, 10414, 02152, and 12145), three genes encoding putative dimethyltryptamine synthases (FFC1_09207, 05684, and 5464), two genes for terpene cyclases, excluding *carRA* (FFC1_00068 and 12027), a P450-1 gene predictably involved in cytokinin biosynthesis (FFC1_079749), and a gene involved in auxin production (FFC1_08093). These results reaffirm a central role for WcoA in the control of a large diversity of SM pathways in *F. fujikuroi*.

**FIGURE 7:**
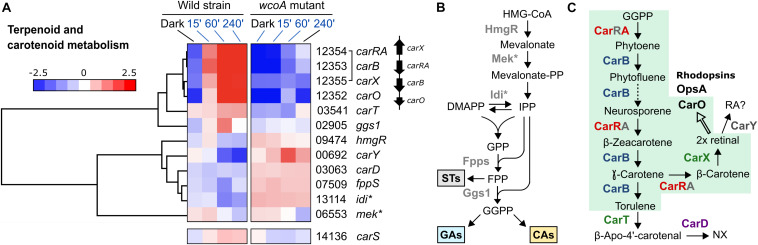
Analysis of the genes involved in carotenoid biosynthesis. **(A)** Hierarchical heatmaps on the effect of illumination and/or the *wcoA* mutation on the mRNA levels of the genes of the carotenoid pathway and heatmap of the regulatory gene *carS*. FFC1_ numbers of the genes, and the names given in the literature, are indicated on the right. The asterisk indicates unpublished functions in *F. fujikuroi*, here inferred from its similarity to enzymes in other species. **(B)** Identification of gene functions in the early steps of terpenoid biosynthesis. STs, sterols; GAs, gibberellins; CAs, carotenoids. **(C)** Identification of gene functions in *F. fujikuroi* carotenoid metabolism. The green area indicates the steps involving proteins of the *car* cluster. The dotted arrow indicates more than one reaction. The thick arrow indicates the use of retinal as cofactor of rhodopsins.

The function of WcoA as a positive regulator was particularly manifest in the case of the four genes of the carotenoid cluster, *carRA*, *carB*, *carX*, and *carO* (Figure 7), which agrees with the previous observations for *carRA* and *carB* in the RT-PCR studies (Figure 1C). The genes *ggs1*, responsible for the formation of the CarRA substrate GGPP, and *carT*, responsible for the cleaving step in NX biosynthesis, were also downregulated in the *wcoA* mutant, and formed a separate group with the genes of the *car* cluster upregulated by WcoA. In contrast, the gene for the final step of NX biosynthesis, *carD*, exhibited an opposite pattern. In this case, WcoA seems to act more as a negative regulator, as has also been observed for three genes for early steps of terpenoid biosynthesis (Figure 7B), *hmgR* (Woitek et al., 1997), *fpps* (Homann et al., 1996), and the gene for the probable isopentenyl-diphosphate delta-isomerase *idi*. The gene *carY* (FFC1_00692), a likely retinoic acid-forming enzyme (Díaz-Sánchez et al., 2016; Figure 7C), exhibited a similar regulation, while the gene for the putative mevalonate kinase (which we call here Mek) is hardly affected by the *wcoA* mutation. Due to its central role in the control of the *car* cluster, the gene for the RING finger protein CarS was included in this study. As previously published, the *carS* gene was induced by light, although not as strongly as the genes of the *car* cluster, and this photoinduction required a functional WcoA protein.

## Discussion

### Influence of Light and WC Proteins on Fungal Transcriptomes

The objective of this work was to investigate the role of WcoA as a regulatory protein in *F. fujikuroi*. WcoA has been described as a key photoreceptor in light regulation in other fungi (Fuller et al., 2015; Corrochano, 2019), and this was its predictable role in *Fusarium*. In addition to *F. fujikuroi*, the effect of the *wcoA* mutation has been investigated in *F. oxysporum* (Ruiz-Roldán et al., 2008), *F. graminearum* (Kim et al., 2014), and *F. asiaticum* (Tang et al., 2020). To check its function in more detail in *F. fujikuroi*, we studied the effect of illumination on the transcriptome of a *wcoA* mutant compared to its wild-type control strain. Previous results on the effect of light on a different wild type showed the occurrence of changes on an extensive number of genes, with a predominance of light-activating effects on light-repressing ones (Ruger-Herreros et al., 2019). Since the *wcoA* mutant was available in a different genetic background, experiments were carried out with its wild-type reference strain, FKMC1995, using the genome annotation available for IMI58289 for more reliable gene assignment. There are significant genomic differences between both strains, as indicated by the finding of 2496 transcripts in FKMC1995 without a clear ortholog in IMI58289.

The effect of light on the transcriptome has been investigated in various fungi with a diversity of techniques (Fuller et al., 2018; Corrochano, 2019). In most cases, light was found to change the mRNA levels of hundreds of genes (see, e.g., microarray assays in *Aspergillus fumigatus*, Fuller et al., 2013, or large-scale cDNA sequencing in *P. blakesleeanus*, Tagua et al., 2020). There are many other studies available on the effect of light on fungal transcriptomes, but here we focus the attention on the reports that included the effect of mutation in *wc* genes. In *Trichoderma reesei*, a close *Fusarium* relative, microarray data showed that 2.8% of the genes were differentially expressed under continuous illumination in relation to a dark control, 55% of them upregulated (Tisch and Schmoll, 2013). Surprisingly, the proportion of genes differentially expressed in the light increased to 9% in the *wc* mutant *blr1*, with a strong predominance of genes repressed by light. The same result was obtained with the mutants of the *wc-2* ortholog *brl2*, indicating that the lack of WCC results in an altered adaptation to constant illumination. A later RNA-seq study in *Trichoderma atroviride* found 246 and 215 genes up- and downregulated after a 30-min light pulse (Cetz-Chel et al., 2016), in total 3.9% of the genes in this fungus (Reithner et al., 2011), but in this case the effect of the mutation of the *wc-1* gene *brl-1* was not investigated. The participation of the WC-1 protein was very different in *Sordaria fimicola*, where the effect of 15 min and 45 min illumination on the transcriptome was checked in the wild strain and in a defective *sfwc-1* mutant (Krobanan et al., 2019). As a result, 874 light-regulated genes were identified, of which 466 lost the photoinduction in the *sfwc-1* mutant, suggesting the participation of another blue-light photoreceptor in light induction of this fungus. Light is less influential in other fungi. In *Ustilago maydis*, only 60 genes were induced and only one was repressed after illumination (Brych et al., 2016), and all of them lost most or all the photoresponse in a mutant of the *wco1* gene. Other fungi, however, are apparently insensitive to light; in the basidiomycetes fungus *Cryptococcus neoformans*, only one gene was significantly affected by illumination (Idnurm and Heitman, 2010).

### Correspondence Between WC Roles in *F. fujikuroi* and *N. crassa*

Studies on WC proteins in *F. fujikuroi* or other fungi benefit from the enormous amount of information accumulated on the functioning of the WC system of *N. crassa*, which is one of the best-known regulatory systems at the molecular level in fungi (Chen et al., 2010b; Corrochano, 2019). A microarray transcriptomic study on the role of the WCC on regulation by light in this model fungus showed that about 5–6% of the genes were differentially expressed during the first 90 min after onset of light, a response largely mediated by the WCC (Chen et al., 2009). Comparison of expression data after eight illumination times, from 5 to 240 min, revealed at least two classes of induction patterns, leading to the classification of genes as early- and late-responsive. Transient responses, with reduction and even recovery of dark mRNA levels after prolonged illumination, are explained by the occurrence of the photoadaptation mechanism mediated by the VVD protein (Corrochano, 2019). A similar photoadaptation mechanism may work in *F. fujikuroi*, in which the VVD ortholog VvdA (Castrillo and Avalos, 2014, 2015) would counteract the activation of WcoA, most likely as a complex with the WC-2 ortholog WcoB. This attenuation mechanism may explain the transient photoinduction observed for many light-regulated genes with fast or intermediate light induction patterns (Figure 2D).

A more precise RNA-seq study in *N. crassa* elevated to 31% the proportion of genes with at least a two-fold change in mRNA levels (Wu et al., 2014). Illumination conditions in this report, with light exposures of 15, 60, 120, and 240 min, facilitate the comparison with our results in *F. fujikuroi*. Different patterns of induction were again observed, in this case separated into four clusters, although the classification as early- and late-responsive genes was conserved. The structural genes of the carotenoid pathway *carB*/*al-1* and *carRA*/*al-2* appear among the 15-top genes with higher early photoinduction (15 min of illumination) [(Wu et al., 2014), genes FFC1_12353 and FFC1_12354 in Supplementary Table 2], consistent with a functional role in protection against light-induced oxidative damage (Avalos and Limón, 2015). Interestingly, three photoreceptors appear in the same top list in both species: Vivid (VvdA, FFC1_04568; VVD, NCU03967), the DASH cryptochrome (CryD, FFC1_04237; CRY, NCU00582), and a photolyase (Phr, FFC1_00478, PHR, NCU08626), indicating that the WC protein coordinates in both fungi other photoresponses. Our data uncovered a possible additional photoreceptor in *Fusarium*, formerly disregarded, also controlled by WcoA. The gene FFC1_12444 encodes a protein with sequence similarity with the phototropin Phot1 of *Arabidopsis thaliana* (Sullivan et al., 2016). Phototropins are serine/threonine kinases that undergo autophosphorylation in response to blue light activation through a LOV domain (Christie et al., 2015). They have been found in algae and higher plants, where they participate in phototropic responses. No FFC1_12444 ortholog was found in the *N. crassa* proteome, and no information is available on biological roles of phototropins in fungi.

In this RNAseq study of *N. crassa* it called the attention the occurrence of a large set of light-repressed genes (Wu et al., 2014). Interestingly, none of them showed early repression (after 15 min light). Similarly, early responsive genes were only found among the light-induced ones in *F. fujikuroi*. WC-1 can also act a as a repressor protein, as recently demonstrated using the photoinducible *al-3* gene as a model, in which a light responsive GATA element was found to be involved in the binding of WC-1 to form a repressing complex in the dark (Brenna and Talora, 2019). However, lack of early gene repression by light in *N. crassa* may be also explained by the action of other negative regulators, which would be activated earlier by WC-1. In addition, the absence of early repression may also be due to the stability of the mRNA already formed prior to illumination, which could mask rapid decreases in transcriptional activity.

It is likely that a central function of WcoA is to modulate the coordinated expression of other regulatory genes, leading to cascade effects. A study of the binding of *N. crassa* WCC to promoters of other genes in its genome revealed that it controls the expression of 24 transcription factors (Smith et al., 2010). This could also be the case for the putative WCC in *F. fujikuroi* with many regulatory genes. An example can be found in FFUJ_01494, ortholog of the WCC-mediated fast induced *csp-1* gene of *N. crassa* whose mRNA is found at lower levels in the *wcoA* mutant (Supplementary Tables 8,10). CSP-1 is a glucose-dependent transcription repressor that mediates the downregulation of 298 clock-controlled genes (Sancar et al., 2011, 2015), and a similar function in *F. fujikuroi* could explain the upregulation of many genes in the *wcoA* mutant. We found 483 orthologs in the FKMC1995 genome for the 298 clock-regulated genes repressed by CSP-1 in *N. crassa* (Supplementary Table 11). However, at least 70% were unaffected by WcoA (Supplementary Figure 5), suggesting differences in the biological role of the *F. fujikuroi* CSP-1 ortholog.

### Light-Independent Roles of WcoA

The extensive changes in the transcriptome of the *wcoA* mutant independent of light are outstanding, even considering the visible phenotypic alterations of its colonies in the dark or under illumination (Castrillo and Avalos, 2015). Such alterations include defective growth, different morphology, lower hydrophobicity, reduced conidiation, and changes in pigmentation and secondary metabolite production (Estrada and Avalos, 2008). Many of these effects are probably due to the lack of a functional WcoA/WcoB complex. However, other light-independent functions could be carried out by WcoA without the involvement of WcoB, as found for the role in pathogenesis of FaWC1 in *F. asiaticum* (Tang et al., 2020). A fraction of the effects of the *wcoA* mutation in the dark could be related to circadian rhythmicity, as suggested by the presence in the *F. fujikuroi* genome of genes for components of a potentially functional clock, including an *frq* ortholog.

Among the great functional diversity of genes affected by the *wcoA* mutation, the most abundant category is secondary metabolism. The production of SMs is subject to different regulations, which generally detect environmental signals, such as pH or stress conditions. As an example, the presence of nitrogen in the medium is a negative regulatory signal to produce gibberellins and bikaverin (Studt and Tudzynski, 2014), but it is a positive signal to produce fusarins (Díaz-Sánchez et al., 2012). In the absence of WcoA, the bikaverin pathway is upregulated, counteracting the presence of nitrogen, but the opposite effect was observed with the genes for GA production. Expression of SM gene clusters frequently involves the participation of different regulatory proteins, and some may be controlled by WcoA. A clear candidate is Sge1 (FFC1_03440), a phosphoprotein homolog to MIT1 from *S. cerevisiae* with known orthologs in other fungi, whose expression is upregulated ca. 10-fold in the *wcoA* mutant. Sge1 was found to regulate positively the production of gibberellins, bikaverin, fumonisin, fusarins, fusaric acid, and apicidin in the IMI58289 wild strain of *F. fujikuroi* (Michielse et al., 2015). This could be additive with other regulatory effects. E.g., transcript levels of FFC1_04144, ortholog of the LaeA-like methyltransferase MrcA of *A. nidulans*, increased in the *wcoA* mutant about two-fold in the dark, and about three-fold and five-fold after 60- and 240-min illumination. Deletion of *mrcA* in *A. nidulans* resulted in a higher production of different SMs and the altered expression of more than 1000 genes (Grau et al., 2019).

Changes in conidiation may be also explained by indirect WcoA effects. The reduced expression of FFC1_06993 in the *wcoA* mutant could be related to its lower conidiation in DG medium (Estrada and Avalos, 2008). FFC1_06993 is homolog of the C2H2 transcription factors LTF2 of *B. cinerea* and Sah-1 of *N. crassa*. In *B. cinerea* LTF2 is induced by light and stimulates conidiation (Cohrs et al., 2016). In contrast, although *sah-1* is induced also by light in *N. crassa* in a WC-dependent manner (Chen et al., 2009), its loss results in enhanced early conidiation (Sun et al., 2012). Moreover, WcoA downregulates the gene for the homeobox protein FFC1_07148, called Htf1 in other *Fusarium* species, where it participates in the development of conidia, especially in the formation of macroconidia (Zheng et al., 2012). It must be noted that *F. fujikuroi* differs from other *Fusarium* species in the regulation of conidiation, as it produces mainly microconidia, while macroconidia are rarely observed.

The lower mycelial hydrophobicity of the *wcoA* mutants of *F. fujikuroi* (Estrada and Avalos, 2008) is a light-independent trait, also described for the *wc1* mutants in *F. oxysporum* (Ruiz-Roldán et al., 2008). To understand the molecular basis of this alteration, we have investigated the effect of the *wcoA* mutation on the orthologous genes for the hydrophobins *Fghyd1-5* of *F. graminearum* (Quarantin et al., 2019). The RNA-seq data showed that *hyd3* was predominantly expressed in our culture conditions, a result that was consistent with the data in *F. graminearum*, where *Fghyd3* also exhibited the highest mRNA content. Light exerted minor changes in *hyd* transcript levels in *F. fujikuroi*, compared to the drastic changes caused by the *wcoA* mutation in some of the *hyd* genes. The reduced hydrophobicity exhibited by the colonies of the *wcoA*/*wc1* mutants could be explained by the strong reduction in *hyd3* transcripts, although it was still the *hyd* gene with the highest expression level. In fact, the analysis of mutants revealed a role for *Fghyd3* in the attachment of hyphae to hydrophobic surfaces in *F. graminearum* (Quarantin et al., 2019).

## Conclusion

WcoA is a master regulatory protein in *F. fujikuroi*, whose activity directly or indirectly affects the expression of more than one thousand genes, involved in a great diversity of cellular processes. This includes the control of most light-regulated genes, presumably in cooperation with the WC-2 ortholog WcoB, as found in other *Fusarium* species (Kim et al., 2014; Tang et al., 2020). However, most of effects are observed irrespective of illumination, indicating that most functions performed by WcoA in *F. fujikuroi* are carried out without the involvement of light detection. Most studies on proteins of the WC protein family have been devoted to light-dependent processes. Our results widen the functional spectrum of WcoA as a representative fungal WC protein that goes far beyond its role as a photoreceptor. This is added to the large amount of data accumulated on the functions of the WCC in *N. crassa* independent of light, controlling the expression of an extensive battery of genes also in the dark, generally related to circadian rhythmicity (Chen et al., 2009; Smith et al., 2010; Wu et al., 2014; Sancar et al., 2015). An important goal in future work with *Fusarium* WcoA should be to understand the basis for its regulatory activity in the dark, including the control of secondary metabolism and its possible connection to circadian regulation.

## Data Availability Statement

The data presented in the study have been deposited in NCBI’s Gene Expression Omnibus repository and are accessible through GEO Series accession number GSE159533 (https://www.ncbi.nlm.nih.gov/geo/query/acc.cgi?acc~=~GSE159533).

## Author Contributions

JP-M performed the experiments. GG and JP-M performed the bioinformatic analyses. MCL and JA supervised the work. JA wrote and edited the manuscript. All authors contributed to the manuscript revision, read, and approved the submitted version.

## Conflict of Interest

The authors declare that the research was conducted in the absence of any commercial or financial relationships that could be construed as a potential conflict of interest.

## Footnotes

1 https://github.com/s-andrews/SeqMonk

2 https://elbe.hki-jena.de/fungifun/
