# Supplementary material for: Impact of the White Collar Photoreceptor WcoA on the Fusarium fujikuroi Transcriptome
Source: Front Microbiol. 2021 Jan 18;11:619474. doi: 10.3389/fmicb.2020.619474 (PMC7871910; doi:10.3389/fmicb.2020.619474)
Supplement: Supplementary file 12 [file Data_Sheet_1.PDF]

Supplementary Material

Role of the White Collar photoreceptor WcoA on the *F. fujikuroi* transcriptome

Javier Pardo-Medina, Gabriel Gutiérrez, M. Carmen Limón, Javier Avalos

A. Effect of 15-min illumination in the wild strain

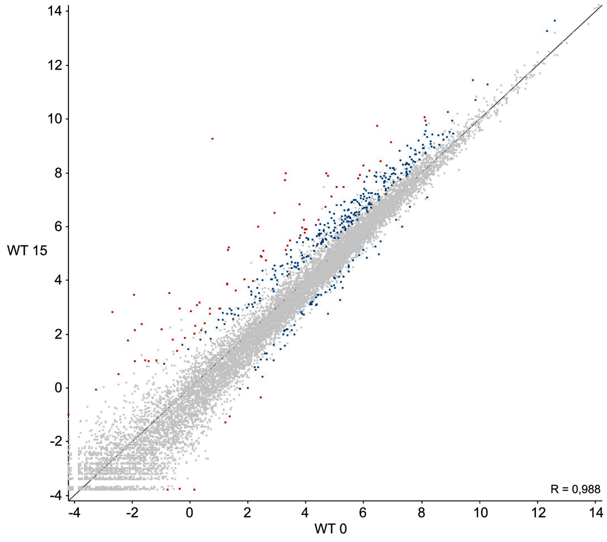

B. Effect of 15-min illumination in the *wcoA* mutant

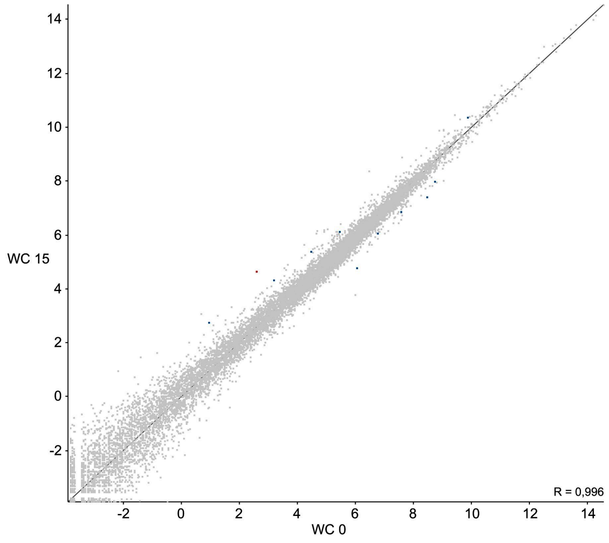

C. Effect of 60-min illumination in the wild strain

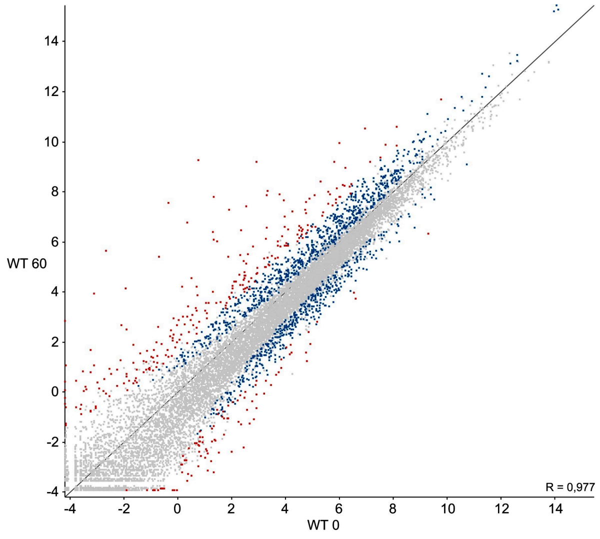

D. Effect of-60 min illumination in the *wcoA* mutant

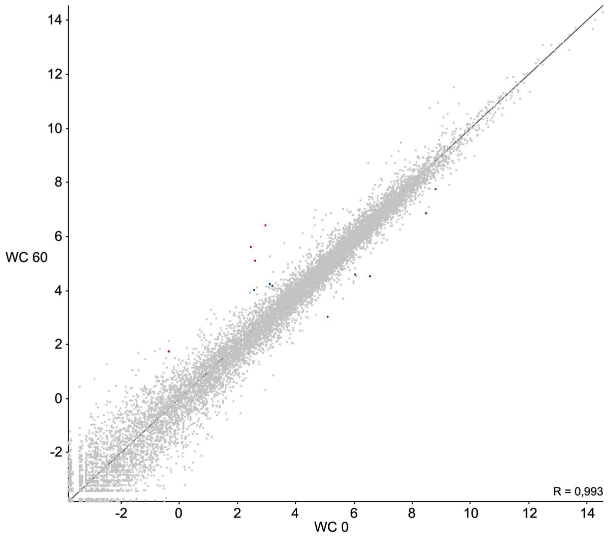

E. Effect of 240-min illumination in the wild strain

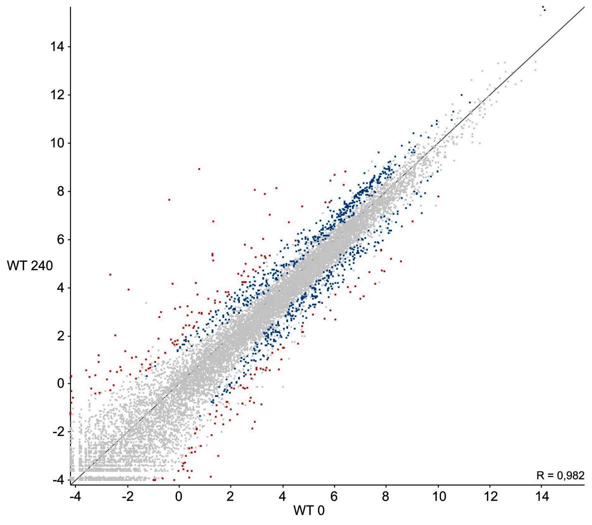

F. Effect of 240-min illumination in the *wcoA* mutant

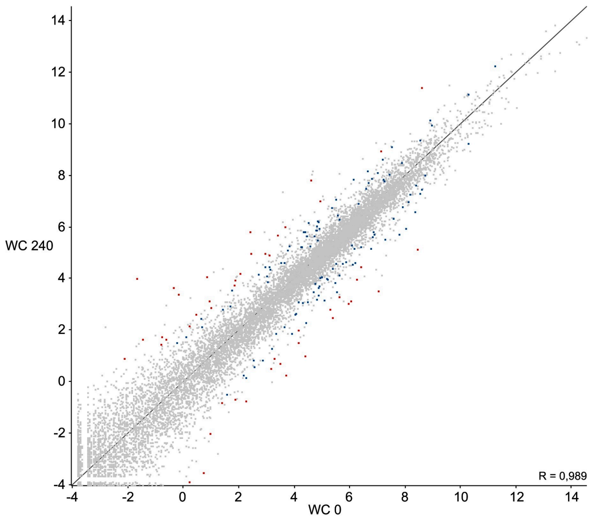

G. Effect of *wcoA* mutation in the dark

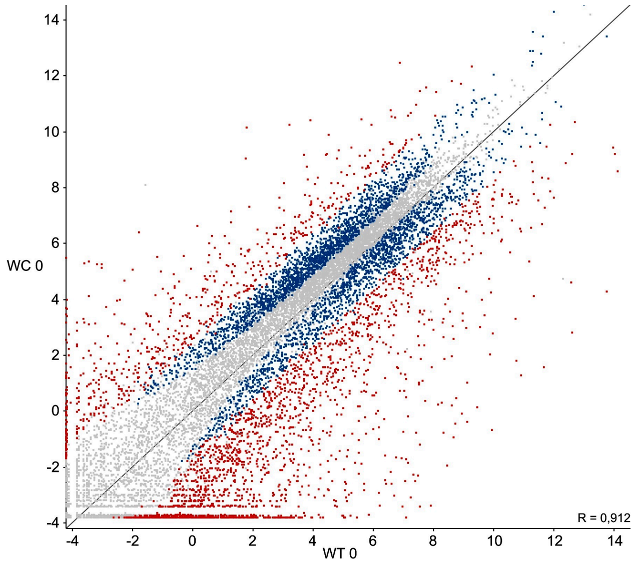

H. Effect of *wcoA* mutation after 15-min illumination

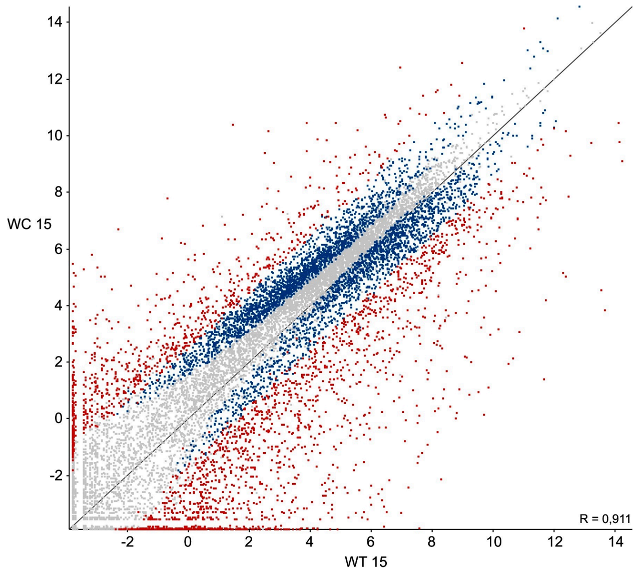

I. Effect of *wcoA* mutation after 60-min illumination

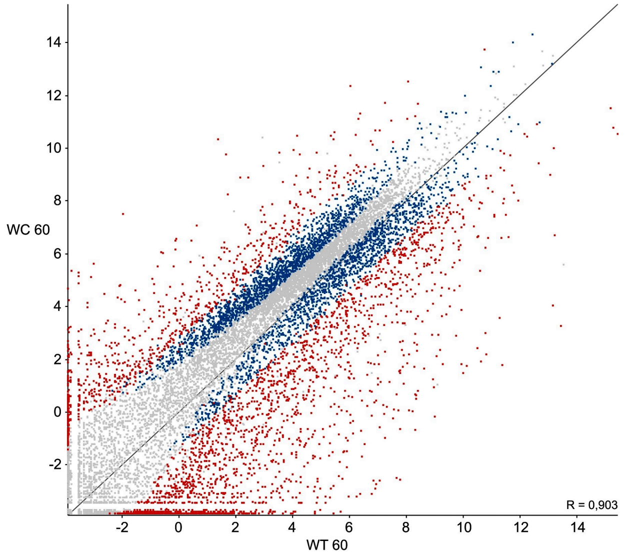

J. Effect of *wcoA* mutation after-240 min illumination

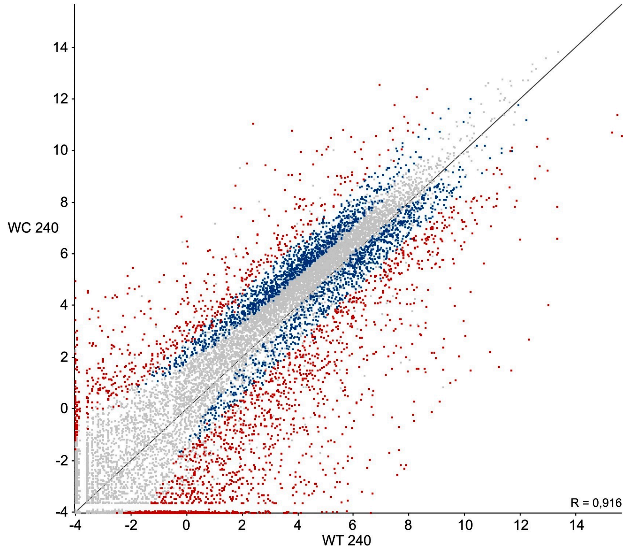

FIGURE S1. Scatter plot representations (log2 rpm) of the effect of light in the wild strain **(A, C, E)** and in the *wcoA* mutant **(B, D, F)**, and the effect of the *wcoA* mutation under different illumination conditions **(G-J)**. Genes differentially expressed according to the Deseq analysis of the Seqmonk program are indicated in blue. Genes exceeding the log2 values of +- 2 are indicated in red.

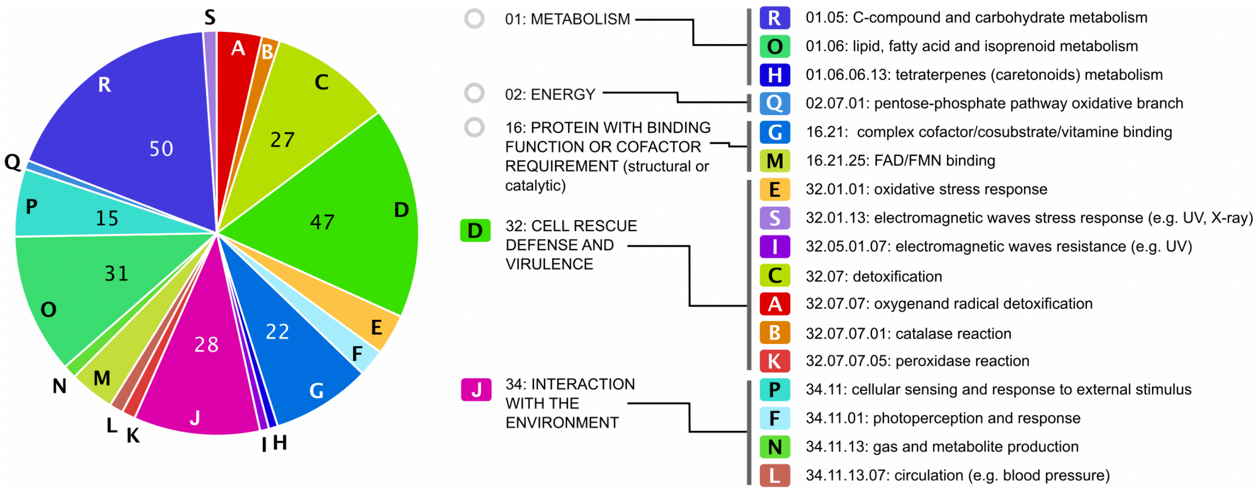

FIGURE S2. Funcat categories of all the genes induced by light in the wild strain of *F. fujikuroi*, irrespective of the illumination time.

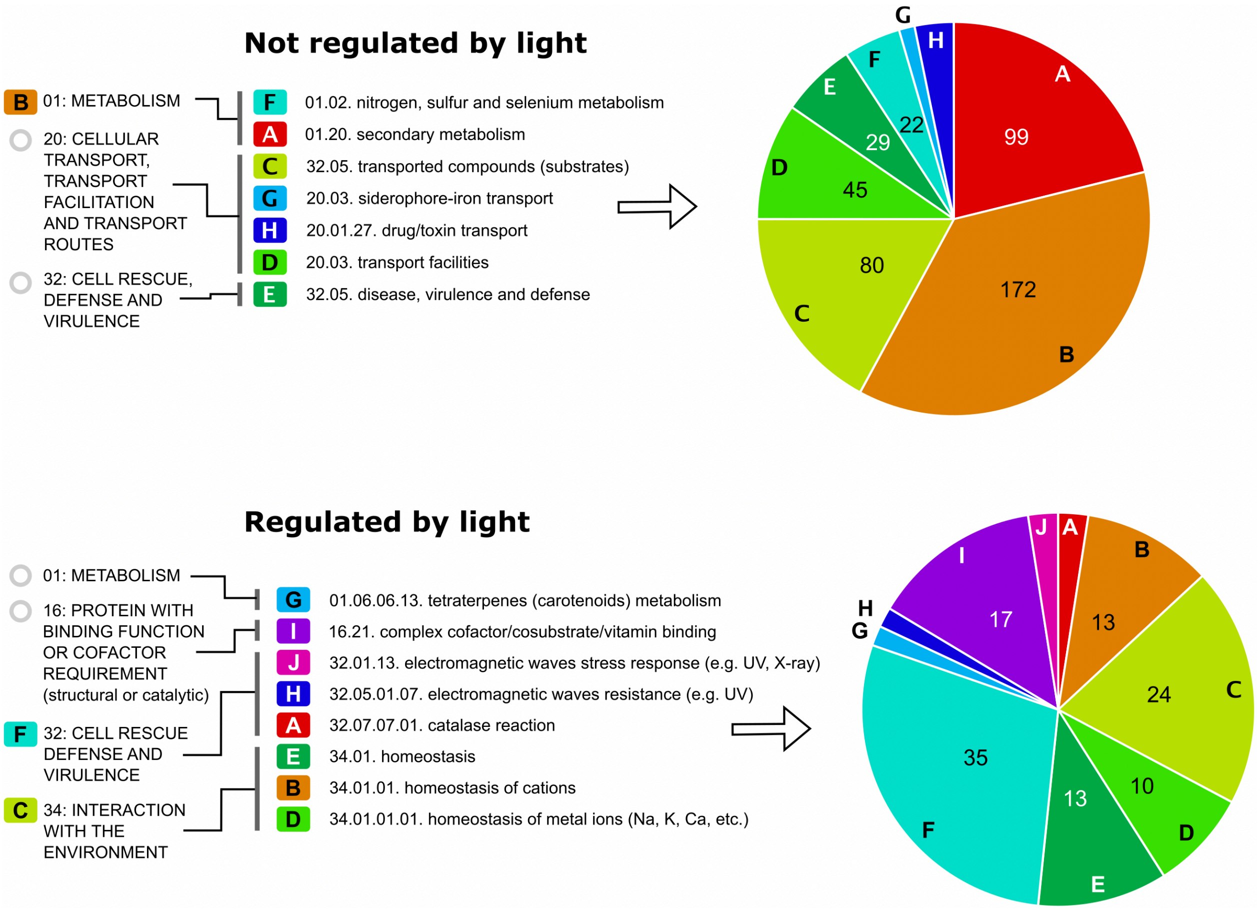

FIGURE S3. Funcat categories of the genes affected by the *wcoA* mutation after application of the SeqMonk intensity difference filter.

A

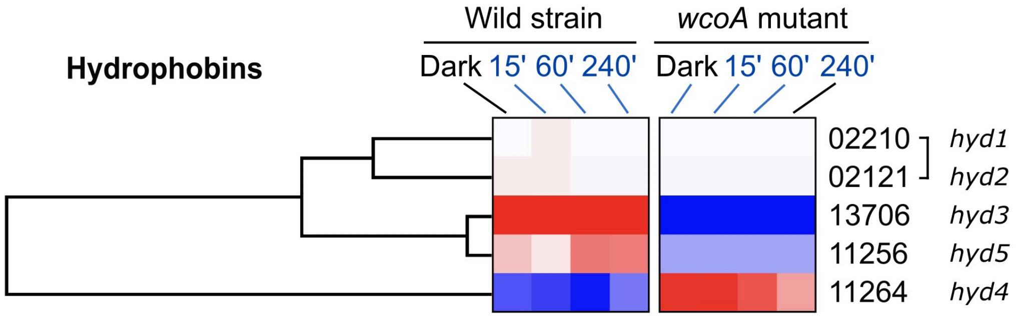

B

Wild strain

Dark	15'	60'	240'	
0	0	0	0	*hyd1*
0.14 ±0.14	0.15 ±0.27	0.05 ±0.09	0	*hyd2*
54706.18 ±21614.77	48440.51 ±23203.65	37838.30 ±17981.42	35048.17 ±20440.23	*hyd3*
1.70 ±1.52	1.16 ±	3.79 ±2.72	3.68 ±2.56	*hyd4*
0.81 ±0.36	0.56 ±0.39	0.12 ±0.20	1.34 ±1.38	*hyd5*

wcoA mutant

Dark	15'	60'	240'	
0	0	0	0	*hyd1*
0	0	0	0.05	*hyd2*
94.85 ±70.61	109.11 ±79.13	106.52 ±113.59	86.16 ±63.13	*hyd3*
0	0	0	0	*hyd4*
27.57 ±7.77	27.08 ±10.94	19.27 ±15.53	10.25 ±4.55	*hyd5*

FIGURE S4. Effect of light and *wcoA* mutation on the expression of the hydrophobin genes *hyd1*, *hyd2*, *hyd3*, *hyd4* and *hyd5*. **(A)** Hierarchical heatmaps on the effect of illumination and/or the wcoA mutation on the mRNA levels of the five genes. (B) Transcript levels (XXX) of the five genes under all the conditions investigated.

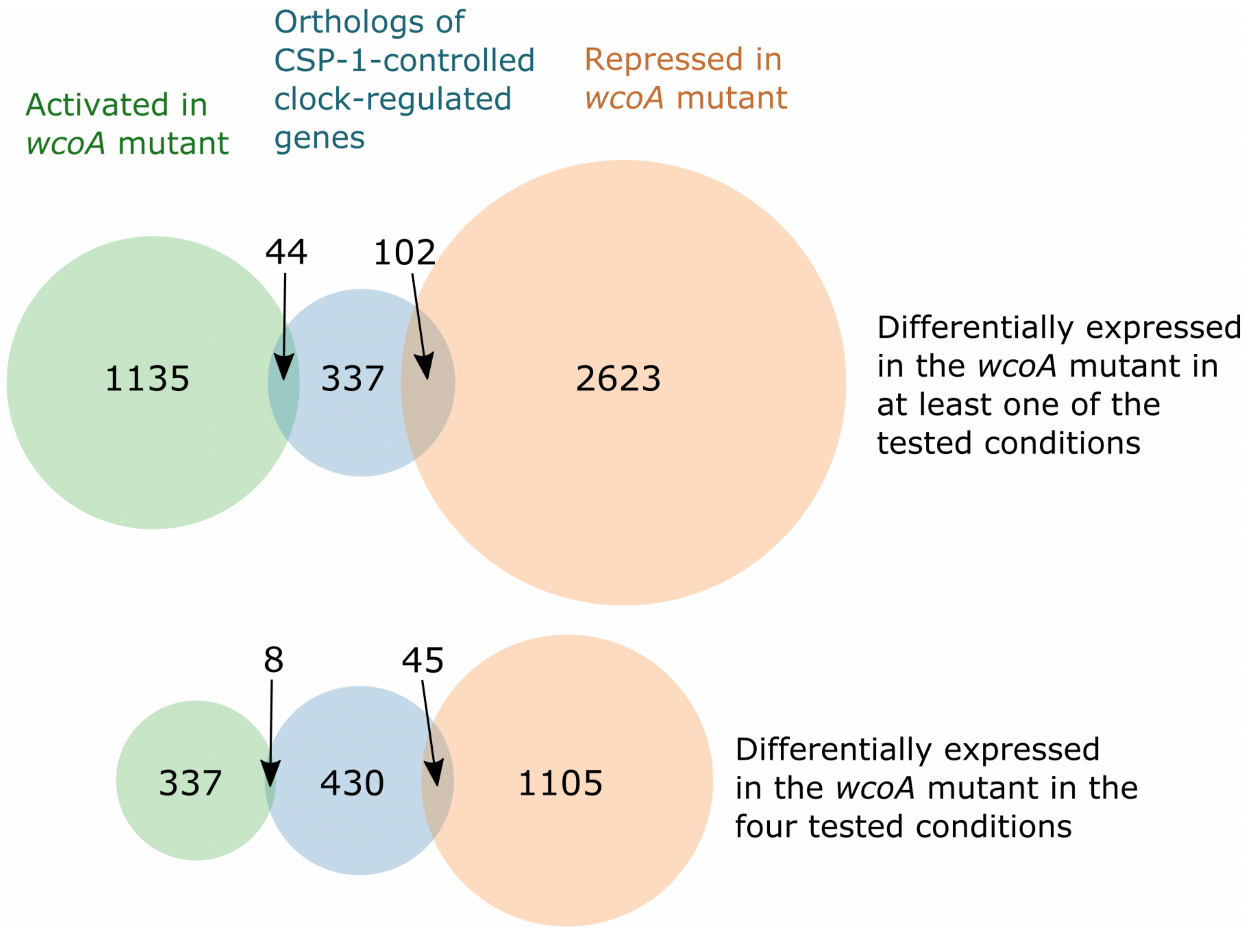

FIGURE S5. Venn diagrams of the overlap between the clock-regulated genes of *N. crassa* controlled by CSP-1 and the genes activated or repressed in the *wcoA* mutant of *F. fujikuroi*. Above: gene sets of *F. fujikuroi* differentially expressed in at least one of the tested conditions (dark, and 15-, 60- or 240-min illumination). Below: gene sets of *F. fujikuroi* differentially expressed in the *wcoA* mutant under all tested conditions.
